# Supplementary material for: Determinants of healthcare insurance coverage among women of reproductive age in Tanzania: A multilevel mixed effect analysis
Source: PLoS One. 2024 Dec 19;19(12):e0302806. doi: 10.1371/journal.pone.0302806 (PMC11658485; doi:10.1371/journal.pone.0302806)
Supplement: Attachment — Submitted filename: Response to Reviewers.docx [file pone.0302806.s001.docx]

Sanun A. Kessy

P.O.BOX 2240

MOSHI, KILIMANJARO

9^th^ November 2024

[sahnunally@outlook.com](mailto:sahnunally@outlook.com)

Dear editor,

Thank you for the opportunity to revise and resubmit our manuscript titled “Determinants of Healthcare Insurance Coverage among Women of Reproductive Age in Tanzania: A Multilevel Mixed Effect Analysis.” (Manuscript ID: PONE-D-24-14959). We appreciate the constructive feedback provided by you and the reviewers, which has helped us improve our manuscript. Below, we address each of the comments raised.

**Editor Comments:**

**Comment 1**: Ensure that all citations are correctly formatted and adhere to the guidelines of journal for article format.

**Response 1:** Thank you for your comment we have ensured that all citations are properly formatted and adhere journal guidelines

**Comment 2**:Add health insurance related policy to your paper to enhance its significance.

**Response 2:** Thank you for your comment, we agree with this comment and we have added the insurance related policy to our paper and we believe it has enhanced its significance.

**Comment 3**:Consult and compare your findings with the most recent studies.

**Response 3:**Thank you for the comment we have noted this and reshuffled our our comparison studies

**Reviewer 1**

**Comment 1:** Please add some data about health insurance coverage for men and children in your country, also what about religion of the studied women does it affect health insurance coverage in Tanzania

**Response 1:** We appreciate your comment, we have added some data on health insurance coverage for men and children in Tanzania, additionally it’s to be noted that religion variable is not captured in Tanzania demographic health survey and hence could not be included in the analysis.

**Reviewer 2**

**Comment 1:** In family/household-based health insurance, access to the health service is provided to family members regardless of age, gender, social status, or religion status. It is more worthy to present the health care utilization for those marginalized groups. Furthermore, access to the drop-out of health insurance and associated factors is important.

**Response 2:** Thank you for your suggestion, in the background section we have included facts that, “The capacity of women in the reproductive age group to attain affordable health coverage and care carries significant societal implications and hinges on the economic well-being of both women and their families. Nevertheless, the accessibility of healthcare services for women in the reproductive age group remains limited in Low- and Middle-Income Countries (LMIC), primarily due to financial”, researches have indicated that women often face a double burden of experiencing a higher prevalence of health issues while simultaneously being primarily responsible for caring for sick children and family members in African context, refer to (<https://www.afro.who.int/sites/default/files/2017-06/report-of-the-commission-on-womens-health-in-the-african-region---full-who_acreport-comp%20(1).pdf> )

Since our study based on secondary data analysis and TDHS does not capture the drop out of health insurance coverage, therefore we have added in the recommendation section for other researchers to explore more on it by both qualitative and quantitative studies.

**Reviewer 3**

**Abstract**

**Comment 1:**Results Section: Please include all methods of analysis used in the study, in addition to the multilevel analysis.

**Response 1:** Thank you for your recommendation, we have added all methods used in the study including multilevel analysis.

**Comment 2:** Conclusion Section: Strengthen the concluding statements and avoid including percentages.

**Response 2:** Thank you for your recommendation, we have strengthened the concluding statement and we have not included the percentages.

**Data Collection Procedure:**

**Comment 3:** The authors analyze DHS data; it would be helpful to provide a summary of the data collection process, outlining the specific steps taken.

**Response 3:** We appreciate your recommendation; we have addressed the concern in Line 133-155 explaining about the data collection process and specific steps taken.

**Comment 4:** Please explain the criteria and sources used to categorize the study variables comprehensively

**Response 4:** Thank you for your concern, the criteria used to categorize the study variables was informed based on the previous literature as comprehensively explained in manuscript in Line 157-166.

**Results**

**Comment 5 & 6:** The analysis in Table 1 contains errors. While the percentage for the "Yes" category (e.g., 5.8%) appears accurate, the percentages for other categories are incorrect. For instance, age-specific percentages should reflect values such as 4.5% among 15–24 years, 5.9% among 25–34 years, and 7.3% among 35–49 years. Kindly reanalyze and update Table 1, revising interpretations accordingly in the results section .Discussion and Conclusion: The discussion and conclusion sections should be revised based on the corrected analysis.

**Response 5 & 6:** Thank you for the observation, We have re-analyzed and updated table 1, lastly, we have revised discussion and conclusion sections based on the corrected analysis.

**Reviewer 4**

**Background:**

**Comment 1:** The citations within the text do not seem to match with the references provided. This seems to be throughout the manuscript. For example, in the third paragraph there is jump from reference number 5 to reference number 14 and 23, 25 in the fourth paragraph. I would like to suggest using reference managing software for proper citation and referencing, throughout the manuscript.

**Response 1:** Thank you for the constructive comment and observation, we have put the citations and they are currently matching and we have used reference manager for citation and referencing throughout the manuscript.

**Methods**:
**Comment 2:**. As the study is the analysis of the secondary information available from the DHS survey, the methodology should be presented as an analysis of secondary information, rather than detailing about the DHS study setting and period, design, data collection procedure and sampling procedure and size (As DHS survey and your study that investigates DHS data are different). The DHS details however can be provided in a single subheading under data source

**Response 2:** Thank you for your comment, as suggested we reshuffle all the sub-titles in the method section and put them under one subtitle named “The data source”.

**Comment 3:** The Demographic and Health Survey is a nationally representative survey (N=15,254) with high efforts gone towards development of variables. Same understanding on the authors’ part is also evident from the mentioning of DHS using the pretested standardized questionnaire within the “data collection procedure” subheading within the manuscript. Why are the variables with original categories in the DHS not used and recategorized then?

**Response 3:** Thank you for your comment and critical observation, some of the variables were used as they were collected from DHS example residence,sex of health of household head among others but some were recategorized for better interpretation based on previous literatures which used DHS for data analysis for easier comparison in discussion section.

**Comment 4:**As the current study also includes the same sample size (15,254), is it possible that, for example married and unmarried categories add to that total (15,254) since the DHS also includes other categories like separated, divorced, widowed, etc. within the Marital status. Also, the references 7-10 do not relate to the statements made.

**Response 4:** We appreciate your comment, marital status had two categories that are married/ in union and unmarried which combined divorced, never in union, no longer living together/separated, living with partner, widowed and hence adding up to the same total, and this is literature based categorization. Lastly we have updated the citation 7-10 and they now relate to the statement made.

**Comment 5:** Assessing potential multicollinearity using VIF is commendable. However, use of variables in the final model must also be reassessed through other criteria when using logistic regression**.**

**Response 5:** Thank you for your comment, during the construction of the final model in our analysis we have employed the Akaike and Bayesian criterion information as stipulated in the line number 184-186. Importantly we used stepwise regression to include the variables in the final model, we have added the statement in our manuscript.

**Results:**

**Comment 6:** Model I, II and III includes variables which do not show significance for the adjusted Odds Ratio. What criterion has been used to include such non-significant variables in the final model. Were they significant when comparing one to one? Kindly go through the below articles and provide necessary corrections or responses.

**Response 6:** Thank you for your comment, We used four(4) criterias to include the non-significant variables in the aforementioned models and these include 1. Literature( possible confounders) 2. Biological plausibility 3. Significance (p-value of less than 0.05) 4. Clinical relevance.

Discussion:
**Comment 7**: This section includes all the findings from the study however the statements made by the authors (besides comparison with findings from other study) may require support from other studies, books, etc. For example: (Line 254-256) “This suggests that having a partner or spouse is beneficial, potentially because of the financial support from being in a dual-income household, which offers more avenues for acquiring health insurance.” Can be rewritten as “Studies have identified that being in a dual income household offers more avenues for health insurance” with citation.

**Response 7:** Thank you for your suggestions, we have rephrased the statement as suggested and we have added the citation to support it, thank you.

**Comment 8:** Discussion, strength and limitation section and manuscript throughout may require some language review and editorial assistance.

**Response 8:**Thank you for your comment**.** We have done some language review to check for spelling and grammar and we have revised accordingly.

**Conclusion:**
**Comment 9**: Are the variables with significant association influencing variables, factors, determinants or else. Kindly revisit the definition and use the same terminology throughout.

**Response 9:** Thank you for your observation, we have revised accordingly and have used the term determinant throughout the manuscript.

**Comment 10:** There may be many ways to empower women economically of which microfinance and income-generating businesses may be some. However, since they were not variables assessed within the study, these cannot directly be quoted into the conclusion for this study. Similar for other findings as well.

**Response 10:** Thank you for your comment, we have revised the manuscript and recommended it according to our analyzed findings.

Thank you again for your valuable feedback. We believe that the revisions have significantly strengthened our manuscript, and we hope it will now meet the standards for publication in Plos One journal.

Sincerely,

Sanun A Kessy

ORCID: https://orcid.org/0009-0007-6854-3887
