# Supplementary material for: Determinants of healthcare insurance coverage among women of reproductive age in Tanzania: A multilevel mixed effect analysis
Source: PLoS One. 2024 Dec 19;19(12):e0302806. doi: 10.1371/journal.pone.0302806 (PMC11658485; doi:10.1371/journal.pone.0302806)
Supplement: Attachment — Submitted filename: Response to Reviewers.docx [file pone.0302806.s002.docx]

Sanun A. Kessy

P.O.BOX 2240

MOSHI, KILIMANJARO

28^th^ November 2024

[sahnunally@outlook.com](mailto:sahnunally@outlook.com)

Dear editor,

Thank you for the opportunity to revise and resubmit our manuscript titled “Determinants of Healthcare Insurance Coverage among Women of Reproductive Age in Tanzania: A Multilevel Mixed Effect Analysis.” (Manuscript ID: PONE-D-24-14959). We appreciate the constructive feedback provided by you and the reviewers, which has helped us improve our manuscript. Below, we address each of the comments raised.

**Reviewer 4**

**Comment 1:** Revisit the editing aspect, throughout the manuscript. Missing “full stops” and other issues present throughout the text.

**Response 1:** Thank you for your feedback regarding the editing and formatting of the manuscript. we have thoroughly revisited the entire document to address all formatting issues, including missing full stops and other punctuation errors

**Comment 2:** Still DHS methodology highlighted in the method section, which can be provided through a citation of the TDHS 2022. Methodology needs to highlight approach to the acquisition of the data, what variables were taken and why, how the variables were recategorized (marital status have more than two categories in the references stated)? The article cited within the manuscript (https://doi.org/10.1186/1475-9276-13-27) can be referred for the methodology section.

**Response 2:** Thank you for your detailed feedback regarding the methodology section.

We have addressed the acquisition of data in the "Data Source" section, starting from line 112, where we have explicitly mentioned the use of the TDHS 2022 dataset and provided the relevant citation. Regarding the selection of variables, we clarified that variables were chosen based on prior literature, as cited within the manuscript (line 156). For the categorization of marital status, we have followed the approach recommended in the literature, including the reference DOI:10.3389/frhs.2022.780550, which provides a detailed framework for recategorization as well as follow your recommendation paper for look up and citation.

**Comment 3:** Also, for the ICC, can it be explained a bit more within the methodology. Have the cluster and strata been considered in the analysis; can it be elaborated?

**Response 3:** Thank you for your feedback. To address the role of the Intra-Cluster Correlation Coefficient (ICC) and clarify the consideration of clustering and stratification in the analysis, we have provided additional details. The ICC was used to quantify the proportion of variation in health insurance coverage attributable to differences between clusters (Primary Sampling Units, PSUs), measuring the similarity within clusters. Higher ICC values indicate greater within-cluster similarity, underscoring the need to account for clustering effects in the analysis. Both clusters (PSUs) and strata were incorporated into the analysis, with PSUs included as random effects to capture hierarchical data structures and between-cluster variation. Stratification variables were used in survey weighting to ensure representativeness and mitigate biases arising from the sampling design. Additionally, all analyses were weighted to account for disproportionate sampling and non-response, ensuring population-level representativeness. These considerations ensured that the complex survey design and hierarchical nature of the data were appropriately addressed.

**Comment 4:** Relating older women with children, education with high paying profession in the discussion may require some basis. For the media part, more than highlighting the country’s internet, focus should be on government and different stakeholders using the media to aware on the health insurance and cite it accordingly.

**Response 4:** Thank you for the valuable feedback. In response, we have written the discussion to ensure it remains evidence-based and concise. Women aged 35 years and older are more likely to subscribe to health insurance due to their increased likelihood of stable employment and caregiving responsibilities, which drive the need for financial risk protection. This explanation aligns with findings from South Africa, Nigeria, and other sub-Saharan African countries. Regarding education, rather than focusing on high-paying professions, we highlight its role in improving individuals’ ability to access and understand health-related information, which facilitates health insurance uptake. Finally, instead of emphasizing internet access, we now focus on how governments and stakeholders can effectively use media platforms to raise awareness about the benefits of health insurance through targeted campaigns. These revisions ensure a concise and evidence-supported discussion.

**Comment 5:** Rewrite the strengths and limitation section. Sounds repetitive and unclear. There may be more limitations as well, one for example can be: unavailability of variables which were significant in other studies etc.

**Response 5:** We appreciate your recommendation; we have addressed the concern and rewrite the strengths and limitation part.

**Comment 6:** The conclusion still has non-related recommendations, that is, information on variables not analysed within the manuscript. Conclusion and recommendations need to be separated. Future studies on drop out from HI suggested in the conclusion which is irrelevant to this study.

**Response 6:** Thank you for the observation, we have re-write the conclusion and recommendations part, as well as separate the conclusion from recommendations as advised

**Comment 7: Include DOIs or weblink in all the references.**

**Response 7:** Thank you for the constructive comment and observation, we have put the citations into proper Plos one format, and they are currently matching, and we have used reference manager for citation and referencing throughout the manuscript.
